# On Tuning the Fluorescence Emission of Porphyrin Free Bases Bonded to the Pore Walls of Organo-Modified Silica

**DOI:** 10.3390/molecules19022261

**Published:** 2014-02-21

**Authors:** Rosa I.Y. Quiroz-Segoviano, Iris N. Serratos, Fernando Rojas-González, Salvador R. Tello-Solís, Rebeca Sosa-Fonseca, Obdulia Medina-Juárez, Carmina Menchaca-Campos, Miguel A. García-Sánchez

**Affiliations:** 1 Departamento de Química, Universidad Autónoma Metropolitana-Iztapalapa, Av. San Rafael Atlixco 186, Vicentina, D. F. 09340, Mexico; 2 Departamento de Física, Universidad Autónoma Metropolitana-Iztapalapa, Av. San Rafael Atlixco 186, Vicentina, D. F. 09340, Mexico; 3 Centro de Investigación en Ingeniería y Ciencias Aplicadas, UAEM, Av. Universidad 1001, Col. Chamilpa, C.P. 62209, Cuernavaca Mor., Mexico

**Keywords:** porphyrin, fluorescence, tetrapyrrole macrocycles, xerogel, sol-gel

## Abstract

A sol-gel methodology has been duly developed in order to perform a controlled covalent coupling of tetrapyrrole macrocycles (e.g., porphyrins, phthalocyanines, naphthalocyanines, chlorophyll, *etc*.) to the pores of metal oxide networks. The resulting absorption and emission spectra intensities in the UV-VIS-NIR range have been found to depend on the polarity existing inside the pores of the network; in turn, this polarization can be tuned through the attachment of organic substituents to the tetrapyrrrole macrocycles before bonding them to the pore network. The paper shows clear evidence of the real possibility of maximizing fluorescence emissions from metal-free bases of substituted tetraphenylporphyrins, especially when these molecules are bonded to the walls of functionalized silica surfaces via the attachment of alkyl or aryl groups arising from the addition of organo-modified alkoxides.

## 1. Introduction

Porphyrins are tetrapyrrole macrocycles. These molecules and their metallic complexes can display diverse and important physicochemical properties. The metal-free bases of porphyrins exhibit intense fluorescence from 600 to 730 nm; the same wavelength range in which a deeper radiation penetration of living tissues occurs and in which the laser beams employed for photodynamic therapy (PDT) operate. For these and other reasons, the entrapment of porphyrins in diverse solids is a subject of the upmost interest. In most cases, porphyrins are physically trapped inside inorganic networks, with no control over the interactions between the macrocycle and the pore network walls. Our main goal will be to covalently trap macrocycles inside xerogel metal oxide networks, while adjusting the interactions of these molecules with the pore surface to achieve a better photoluminescence display.

The central heme group present in either hemoglobin or cytochrome is an iron porphyrin [1]. This group is responsible for O_2_, CO_2_, and NO_2_ transport and the occurrence of numerous metabolic functions. The structure responsible for the capture and employment of solar radiation in chlorophyll is a chlorin, which is similar in structure to porphyrin. The porphyrin in the heme group of blood and cytochrome, the chlorin in chlorophylls, the corrins in B_12_ vitamin, and similar natural structures are all members of the tetrapyrrole macrocycle family [1].

During the last century, synthetic tetrapyrrole macrocycles, including porphyrins (H_2_P), phthalocyanines (H_2_Pc), and naphthalocyanines (H_2_Nc) were synthesized. Both natural and synthetic tetrapyrrolic macrocycles have an extensive delocalized π-electron system that confers them high chemical and thermal stabilities [2]. These compounds display other interesting physicochemical properties that allow their application in technological fields such as catalysis [3,4], electrochemistry [5], optics [6], and sensoring [7,8].

**Figure 1:**
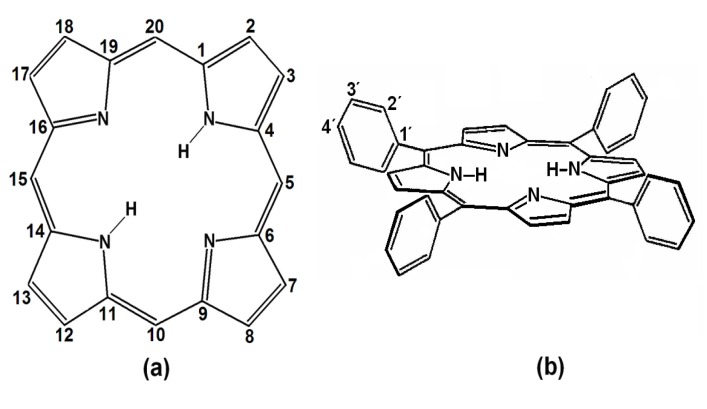
(**a**) Structure and peripheral positions of atoms in a porphin macrocycle (H_2_P) and (**b**) chemical structure of 5, 10, 15,20*-meso-*tetrakisphenylporphyrin (H_2_TPP).

Porphyrins are porphin derivatives (Figure 1a), formed by four pyrrole rings connected by methine (=CH-) groups. This structure includes two pyrrolic (*i.e.*, possessing hydrogen) and two pyrrolidinic nitrogens at the center of the macrocycle, which participate in the macromolecular electron system resonance. These four nitrogens allow porphyrins to act as strong divalent and tetracoordinated ligands, which permits the formation of metallic porphyrinic complexes with practically all the metallic elements of the periodic table [9,10].

*meso*-Porphyrins are porphine derivatives with one or several of their methinic hydrogens substituted by alkyl or aryl groups, such as phenyls, as in 5,10,15,20-*meso-*tetrakisphenylporphyrins (H_2_TPP) (Figure 1b). Furthermore, diverse substituents can be attached at the *ortho-, meta-* or *para-* positions (*i.e.*, positions 2', 3' or 4') of these peripheral phenyls through classic electrophilic or nucleophilic aromatic substitution reactions (*i.e.*, S_E_Ar or S_Nu_Ar), thus producing substituted tetraphenylporphyrins, H_2_T(*S*)PP. The inclusion of organic functions on the periphery of porphyrins provides them with a notable chemical versatility when incorporated into more complex structures, something which enables a great number of applications [3,4,7,8,11,12,13,14,15,16,17].

Many porphyrin free bases (*i.e.*, the corresponding unmetalled species) exhibit a red fluorescence in the 600 to 800 nm range [9,10,18] and that red or infrared light (wavelengths ranging from 600 to 1,500 nm) achieves a better penetration into tissues [10,11]. Due to these properties, and since some free bases of substituted porphyrins are selectively adsorbed by malignant cells or diverse bacteria [19,20,21], compounds such as H_2_T(*S*)PP have been used to kill cancer cells or microorganisms through the so-callled photodynamic therapy (PDT) technique, which is based on the toxicity of singlet oxygen (^1^O_2_) species generated by the incidence of red laser light on the porphyrin (or other compounds named photosensitizers) that are selectively deposited on malignant cells [9,10,20,21,22,23].

In order to exploit the above properties and those of similar organic structures, it is necessary to trap or fix these macrocycles within organic or inorganic solid supports. The sol-gel method (considered soft chemistry or *chimie douce*) [24,25], allows the insertion of chemical species that extend from simple cations [26] to organic molecules [27], biochemical molecules [28,29], and even cells and bacteria [30,31]. One key factor of the sol-gel procedure is to use organometallic precursors, such as metal alkoxides, M^z+^(OR)_z_ to react with water through hydrolysis and polycondensation reactions to generate an intricate tridimensional pore network constituted by M-O-M bonded species and remnant M-OH groups. These last groups can be eliminated through thermal treatment to create the final inorganic oxide xerogel matrix (M^z+^O_z/2_).

Our research group has been pursuing an optimal mimicking of the physicochemical properties that tetrapyrrolic species display in solution, once these molecules are trapped within translucent xerogel inorganic metal oxide matrices. The key factor for achieving this goal consists in either physically (*i.e.*, van der Waals forces) or chemically (*i.e.*, covalent bonds) binding these molecules inside the pores of inorganic networks synthesized by the sol-gel method. The first stage of this process consists in the determination of the optimum molar composition of the gel mixture that can render optically translucent monolithic silica xerogels. To do this, *μ-*hydroxyaluminum tetrasulfophthalocyanine, (OH)AlTSPc was selected as a spectroscopic tracer to be physically trapped inside the pore network. It was found that a 19.6:1:10^−3^:10^−3^–10^−6 ^H_2_O:Si(OC_2_H_5_)_4 _(or TEOS):HCl:(OH)AlTSPc molar mixture provides translucent silica xerogels with the macrocyclic species trapped inside the pores [32]. By using the same molar ratios, it was possible to physically trap in stable (*i.e.*, monomeric and intact) form not only (OH)AlTSPc species, but also diverse tetrasulfophthalocyanine metal complexes (MTSPc; M = Fe, Co, Ni, Cu, Eu, or Sm) [33,34], lanthanide-tetraphenylporphyrins, Ln(ac)TPP·2S (S being a solvent) [35], as well as free bases of substituted tetraphenylporphyrins, H_2_T(*S*)PP [36,37].

It was found that the pore widths created around a trapped macrocycle depended on its structure and on the identity of the cation present in the respective metallic complex. Nevertheless, the coordination capacity and fluorescence that macrocyclic species show in solution are inhibited or can even disappear when trapped in a solid network. This effect can be attributed to the interaction between the the macrocycles and remnant Si-OH groups that are attached to the pore walls. To inhibit the above effects, some strategies were tested [38]: (i) to situate the macrocycle far from the pore walls by establishing simple or long bonds [37,38] (by means of functionalized silicon alkoxides, FA); (ii) to substitute the surface hydroxyl groups by alkyl or aryl groups coming from organosubstituted alkoxides (OSA) [39]; (iii) to trap or fix the macrocycle inside networks made of diverse cations (ZrO_2_, TiO_2_, Al_2_O_3_, *etc*.) or (iv) combinations of these previous options [38].

At the first instance, the physical trapping of H_2_T(*S*)PP species inside the pores of translucent silica xerogels was performed by means of tetraphenylporphyrins including –OH, NH_2_, and -COOH substituent groups at the *ortho-* or *para*-positions. Through this option, porphyrins were trapped in stable and monomeric form, however, the fluorescence was only partially preserved in the case of *ortho*-amine-substituted porphyrins, H_2_T(*o-*NH_2_)PP [36]. To establish covalent unions with the pore walls, it is necessary to synthesize the respective precursors through the union of the organic function present in functionalized alkoxides (FA) and the appropriate groups attached to the substituted tetraphenylporphyrins, H_2_T(*S*)PP. To obtain monolithic translucent xerogels with H_2_T(*S*)PP species bonded to the network, the precursor was added to a gelling mixture and the ensuing hydrolysis and polycondentation reactions of alkoxide groups allowed the integration of the macrocycle into the gelling network. Though this last procedure, fluorescence was better preserved by using H_2_T(*p*-COOH)PP species, but some interference with the silica network was also observed [37]. Long unions could be established when the use of functionalized alkoxides and polymer precursors, such as diamines, diacids, and lactams was combined. For acheiving a better preservation of fluorescence, the average pore sizes obtained through this option should be set larger, thus suggesting the possibility of modulating this parameter by the use of macrocyclic species [38].

The option of substituting surface *S*-OH groups by alkyl or aryl groups, was explored by using again (OH)AlTSPc species as a probe which could be physically trapped inside the pores of organosubstituted silica. In this way, it was determined that the addition of less than 3% v/v of organo-substituted alkoxide (OSA) produces translucent monolithic xerogels [39]. In a second set of probing experiments, the cobalt tetrakis-(*para-*carboxyphenyl)-porphyrin, CoT(*p-*COOH)PP was bonded to the pore walls of organosubstituted silica. It was found that the absorbance of UV-Vis bands, in the respective spectra of these samples, depended on the length of the alkyl chain attached to the silica pore walls. This result hinted at the possibility of decreasing the population of the Si-OH groups and tuning the polarity inside the pores as a means for optimizing the display of the luminescent properties of the species trapped inside the network [40].

Recently, this last methodology was successfully applied to preserve the fluorescence that chlorophyll displays in solution by fixing this molecule inside the pores of organosubstituted silica and to SBA-15 mesoporous silica [41].

In this document, we present the first results of a mixed strategy that consists of covalently bonding the H_2_T(*p*-COOH)pp and H_2_T(*o*-NH_2_)PP species (*i.e.*, those that showed the better results in the previous experiments) inside the pores of organomodified silica, as a way to reduce the interactions with the pore walls and tune the polarity inside the pores of the network thus optimizing the fluorescence of the trapped species.

## 2. Results and discussion

### 2.1. Absorption and Fluorescence Characterization of Porphyrins in Solution

The electronic transitions of porphyrins display an extreme sensitivity toward structural modifications and changes in the polarity of the surrounding environment [9,10,18]. For this reason, the absorbances and emissions radiated by these species can be monitored in order to analyze the physicochemical state of trapped species inside the pores of organo-modified silica obtained from the sol-gel process and, to infer the structural modifications of the trapped macrocycle in terms of its surrounding polarity. Absorptions can be attributed to π–π* electronic transitions; the most intense band of which corresponds to a Soret band that is displayed in the range extending from 410 to 440 nm is assigned to the a_2u_→e_g _(transition with intervention of the free electrons pairs of the pyrrole nitrogens [9,42]. The Q bands (Q_IV_ to Q_I_), observed in the range from 500 to 700 nm and assigned a_1u_, a_2u_→e_g _(transitions are due to charge transference from the pyrrole carbons to the other atoms of the macrocycle [10,42]. These bands are weakly influenced by the nature of the cation in the respective complexes or by the presence of peripheral groups in the substituted porphyrins.

**Figure 2:**
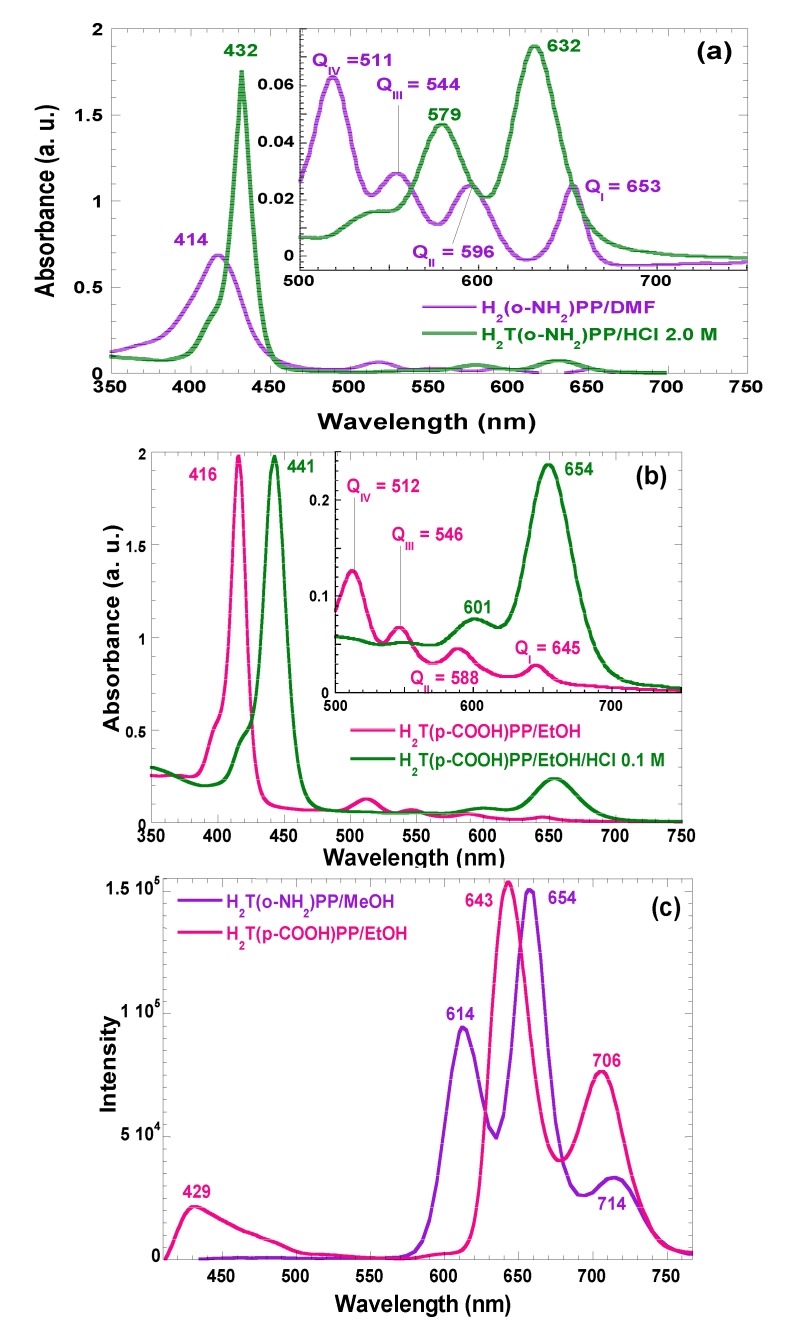
Absorption spectra in solution (organic or acidic media) of the (**a**) H_2_T(*o-*NH_2_)PP in methanol and (**b**) H_2_T(*p-*COOH)PP species in ethanol. (**c**) Fluorescence spectra of the same species in organic solution and with λ_ex_ = 370 nm. All spectra are taken at 25 °C.

In the absorption spectra of synthesized and purified porphyrins that are involved in the present investigation, characteristic bands of neutral free bases in solution can be observed (Figure 2). For the H_2_T(*o-*NH_2_)PP species the Soret band appears at 414 nm, while assorted Q bands are displayed at 511, 544, 596, and 656 nm (Q_IV_ to Q_I_, respectively) (Figure 2a). Similarly, in the case of the spectra of H_2_T(*p-*COOH)PP species, the Soret band was observed at 416 nm while Q bands appear at 512, 446, 588, and 645 nm (Figure 2b). However, under acidic conditions, the Soret bands were red-shifted to 432 and 443 nm for H_2_T(*o-*NH_2_)PP and H_2_T(*p-*COOH)PP species; Q_II_ and Q_I _bands appear at 579 and 632 nm in the spectrum of the first species and at 601 and 654 nm for the second porphyrin. This second signal pathway is due to the formation of the respective protonated dicationic H_4_T(*o-*NH_2_)PP^2+^ and H_4_T(*p*-COOH)PP^2+^ species, whose existence is evident due to the change of the solutions to a green color. As it was mentioned in the introduction section, many metal-free bases of porphyrins show intense fluorescence in the red region of the visible spectrum [9,10,18]. In the fluorescence spectra (Figure 2c) of H_2_T(*o-*NH_2_)PP species in solution, three bands were observed along the red region, *i.e.*, at 614, 654, and 714 nm, as well as two bands at 643 and 706 nm, for the case of H_2_T(*p*-COOH)PP species.

### 2.2. Covalent Bonding of Porphyrins to the Silica Pore Network

**Scheme 1:**
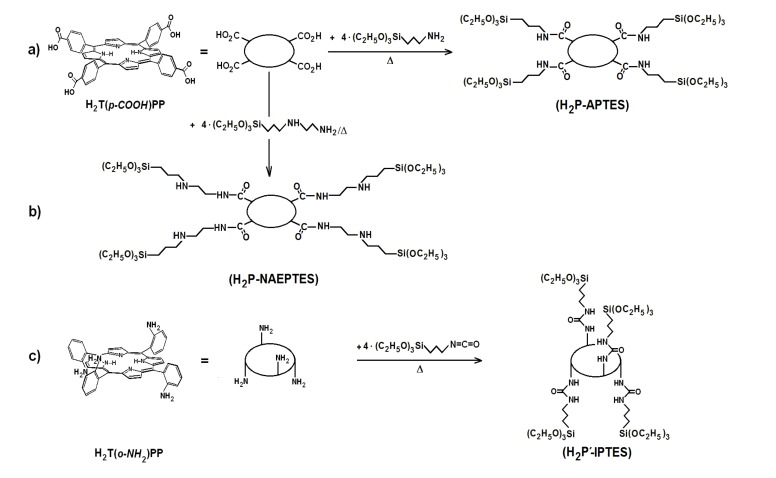
Synthesis route for the obtainment H_2_P-FA precursorsfrom H_2_T(*p-*COOH)PP and H_2_T(*o-*NH_2_)PP species and the following functionalized alkoxides: (**a**) APTES, (**b**) NAEPTES and (**c**) IPTES.

**Scheme 2:**
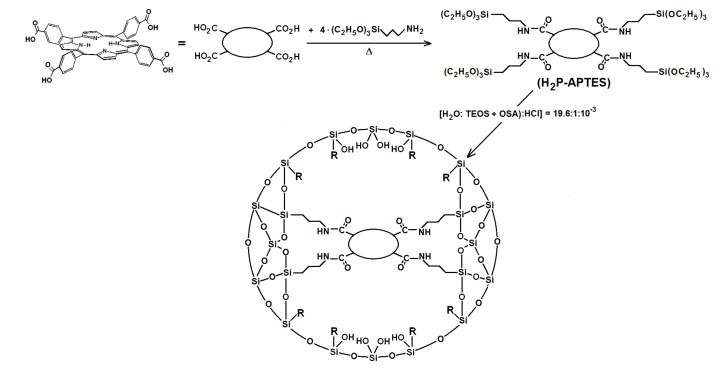
Synthesis route for the covalent union between substituted tetraphenylporphyrin metal-free bases and the pore surface of xerogel networks of organo-modified silica. H_2_P-APTES represents the precursory species and R is an alkyl or aryl group arising from the use of organosubstituted alkoxides.

As stated in previous paragraphs of the introduction section, the stability and luminescent properties of tetrapyrrole macrocycles trapped inside metal oxide networks, synthesized by the sol-gel method, were better preserved when these species were covalently bonded to the pore network. These chemical bonds can be established by only using functionalized alkoxides, or by combinations of them with polymeric precursors, such as diamines, diacids, lactams, *etc.*, which produce longer bonds [37,38]. However, through this last procedure, some of the fluorescence inhibiting interaction between the macrocycle molecules and the surface S-OH groups still subsists. As postulated above, the next step is to explore a combined strategy consisting of establishing covalent unions with the network and to tune the polarity inside the pores and around the trapped macrocycle species by substituting the S-OH groups for Si-R moieties, where R represents an alkyl or aryl group arising from the use of organosubstituted alkoxides (OSA) [39,40,41]. The first step to perform this substitution consists of synthesizing the required precursors (H_2_P-FA) from the union between porphyrin substituents and organic groups present in the adequately functionalized alkoxide (FA). To obtain the precursor from the H_2_T(*p-*COOH)PP species, the respective amide bonds can be obtained from the reaction of carboxyl groups (-COOH) and amine groups (-NH_2_) of the 3-aminopropyl-triethoxysilane (APTES) or 3-*N*-(2-aminoethylamino)propyltrimethoxysilane (NAEPTES) (Scheme 1). In the case of the H_2_T(*o-*NH_2_)PP species, the precursor is obtained from the formation of urethane (-NH-CO-NH-) moieties created by the nucleophilic addition of porphyrin amine groups to the isocyanate groups (-N=C=O) of the 3-Isocyanopropyltriethoxysilane (IPTES) (Scheme 1). The formation of amide or urethane unions was proved and followed through FTIR spectroscopy as previously reported [37,38,39,41]. In a second step the translucent and monolithic organo-modified silica xerogels, with the H_2_T(*p-*COOH)PP or H_2_T(*o-*NH_2_)PP species bonded to the pore network were synthesize from molar ratio mixture equivalent to 19.6:1:10^−3^:10^−3^–10^−6^ of water, triethoxysilane (TEOS), acid catalyst and the respective precursor (Scheme 2), which rendered a final V_f_ total volume [24,25,26,27,28]. The diverse organosubstituted alkoxides (OSA) were included in the mixtures as the 1.0 % v/V_f_.

### 2.3. Absorption and Fluorescence Spectra of Porphyrins Bonded to Organo-modified Silica

**Figure 3:**
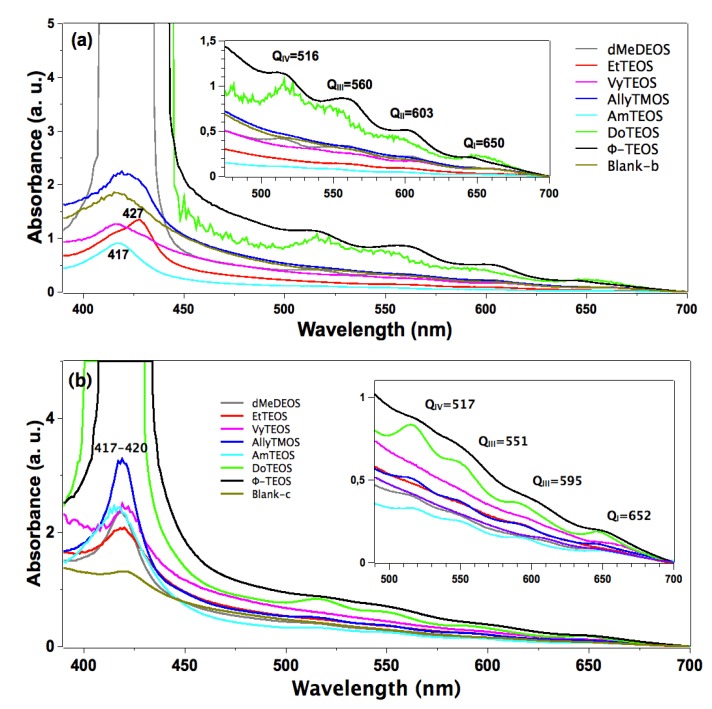
Absorption spectra of the H_2_T(*p-*COOH)PP species covalently bonded, through the use of (**a**) APTES or (**b**) NAEPTES, to the pore walls of silica modified with organic groups coming from the use of 1% v/V_f_ of OSA.

In the absorption spectra of the set of samples in which APTES was used as a bridge to establish the bond connections of the macrocycle with the organomodified silica, the characteristic Q band pathway at around 516, 560, 603 and 650 nm can be observed (Figure 3a). In these spectra, the Soret bands appear with different absorbances and around 417 nm. The pathway of signals observed in all the cases can be associated with the stable free base of H_2_T(*p-*COOH)PP, but immersed in a solid medium. Furthermore, the very different absorbances displayed by these bands can only be associated to the existence of a different physicochemical environment around the macrocycle. Since all samples were synthesized from the same molar mixture, with the exception of the identity of the alkyl or aryl group in the organosubstituted alkoxide (OSA) involved, the differences in absorption observed in the UV-Vis spectra of the final xerogel samples can be only attributed to the existence of these groups in the solid samples. In this set of samples, the Soret bands absorbances follows decreasing sequence: phenyl > dodecyl > blank-b >allyl > methyl > ethyl >vinyl > amyl. As it was mentioned in the Experimental section the reference sample, named blank a, was synthesized from the same molar ratio but, with neither OSA compounds nor bonded porhyrins. Additionaly, blank-b and blank-c reference samples were synthesized by bonding the porphyrin molecule to the silica pore walls through APTES or NAEPTES, respectively.

In the case of the samples synthesized using NAEPTES as the connection molecule, the absorption spectra show Soret bands at around 417–420 nm and Q (IV to I) bands at 517, 551, 595 and 652 nm, respectively (Figure 3b). In all cases the pathway of signals can be associated with the existence of stable (non-protonated) free bases of H_2_T(*p-*COOH)PP. Contrasting with those samples obtained through the use of APTES, the bands observed in the spectra of the xerogels synthesized using NAEPTES were more evident. Additionally, in this set of samples the absorbances decrease according to the following sequence: phenyl > dodecyl > allyl > vinyl > amyl > blank-c > ethyl > methyl. All these samples sets were translucent, although some of them resulted in somewhat yellowish monolithic xerogels without visible signals of the presence of precipitates or heterogeneities.

**Figure 4:**
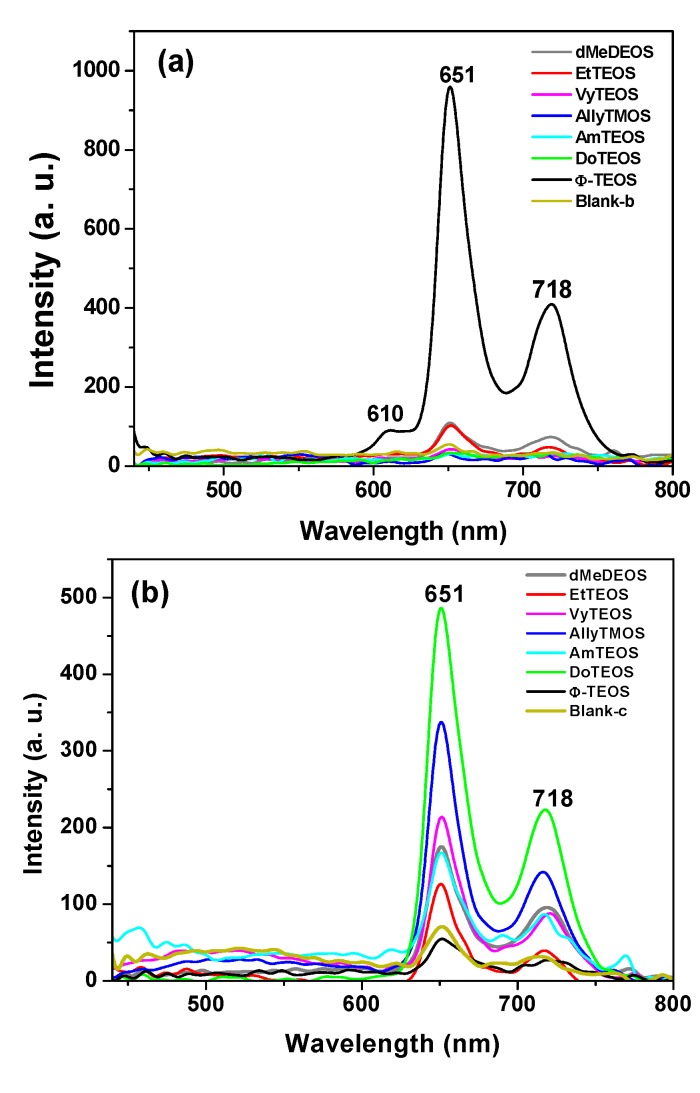
(**a**) Fluorescence spectra (λ_exc_ = 420 nm) of the H_2_T(*p-*COOH)PP species covalently bonded, through the use of APTES or (**b**) NAEPTES, to the pore walls of silica organically modified with groups resulting from the use of 1% v/V_f_ of OSA.

In the fluorescence spectra of organo-modified silica xerogels containing the H_2_T(*p-COOH*)PP species bonded through the use of APTES, two principal bands appear at 651 and 718 nm (Figure 4a). These bands are observed red shifted with respect to those observed in solution (*i.e.*, at 643 and 706 nm in Figure 2c), which could be attributed to the different polarity existing inside the silica pores and interactions with the surface groups. However, there is a drastic intensity difference between the sample modified with phenyl groups (Φ-TEOS) and the other samples. This difference is attributed to the interactions of the porphyrin macrocycle with the groups attached in the pore walls. That is, when using APTES as a bridge and way to separate the porphyrin and the pore walls, the best result was obtained when phenyl, methyl or ethyl groups existed on the surface of the pores. The presence of such groups leads to lower polarities, which make the occurrence of the light emission process of the porphyrin easier.

The very same two bands mentioned above were observed at 651 and 718 nm in the fluorescence spectra of the final xerogels in which NAEPTES was used to bond the porphyrin to the pore walls of organomodified silica (Figure 4b). In this set of samples, the intensities of the signals were similar to each other and apparently depended on the identity of the organic group that was present on the surface of the pores. As mentioned before, the existence of these organic groups on the internal surface of the pores formed around the bonded porphyrin induces a lower polarity inside the voids, which facilitates the occurrence of the fluorescence process of the macrocycle. Higher fluorescence intensities were determined when these groups were dodecyl (DoTEOS), allyl (AllyTEOS), vinyl (VyTEOS), and methyl (dMeDEOS).

The contrast between the two sets of synthesized samples, shows that a short bridging bond resulting from the use of APTES gives the best results, which therefore occur in the presence of compact organic substituents such as phenyl, methyl or ethyl groups. On the other hand, when long unions, created by the use of NAEPTES were established, the best results were observed in the presence of long alkyl chains, such as dodecyl or those that possess double bonds, such as allyl or vinyl groups. Due to their size, methyl groups attached to the silica pore walls remain around the porphyrin and suffer no great interaction with it. A similar event occurs with other short chains, such as ethyl, vinyl or allyl groups that could remain relatively close to the porphyrin macrocycle. However, longer chains such as dodecyl or phenyl groups could be interacting strongly with the porphyrin conjugated system while staying closer to it. These observations suggest that the proximity, localization, and identity of these surrounding groups induce a physicochemical environment around the macrocycle that facilitates the occurrence of the fluorescence emission of porphyrin, in a similar way as this molecule displays in solution.

### 2.4. ^29^Si-NMR and SEM Characterization

**Figure 5:**
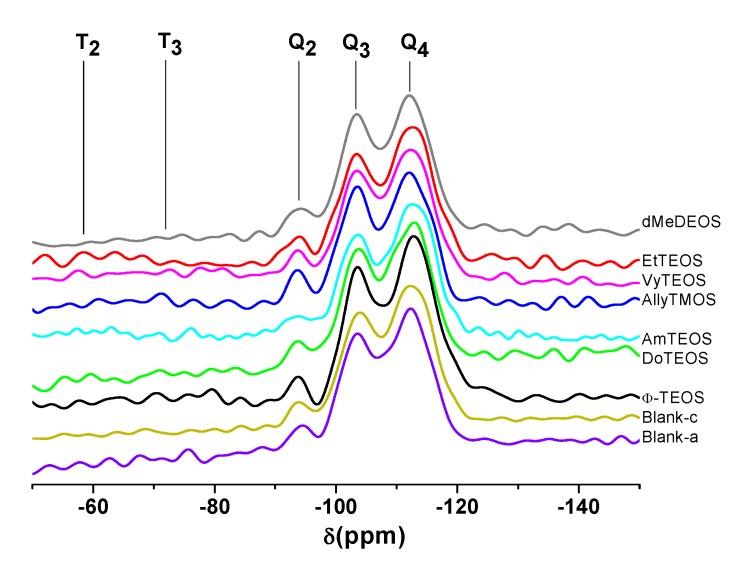
HPDEC ^29^Si-RMN spectra of the xerogels with the H_2_T(*p-*COOH)PP species covalently bonded, through the use of NAEPTES, to the pore walls of silica organically modified with organic groups coming from the use of 1% v/V_f_ of OSA.

The existence of alkyl or aryl groups attached to the structure of silica can be proved through ^29^Si- NMR spectroscopy. For instance, in the respective HPDEC ^29^Si-NMR spectra of samples synthesized using NAEPTES as linking species (Figure 5), the Q_2_, Q_3,_ and Q_4_ bands can be attributed to silicon atoms bonded to two, three or four siloxane chains (–O–Si–) and surface silanol groups (Si-OH) in order to complete four unions, respectively [43,44]. Additionally, the low intensity bands localized at δ = −60 and −90 ppm can be attributed to the existence of silicon atoms bonded to alkyl or aryl carbon groups, two (T_2_) or three (T_3_) siloxane chains, and surface hydroxyl groups that are required for completing four unions [43,44]. In the analyzed set of spectra, the intensity of the signals followed the decreasing sequence Q_4_ > Q_3_ > Q_2_, thus suggesting the existence of a highly condensed silica network principally constituted by silicon atoms bonded to four siloxane chains. However, the presence of Q_3_ and Q_2_ bands reveals the existence of a substantial amount of silicon atoms linked to one hydroxyl group and a lower population of these atoms linked to two hydroxyls. Besides, the existence of T_2_ and T_3_ bands, which depicted a low intensity, confirmed the presence of alkyl or aryl groups attached to the silica network.

**Figure 6:**
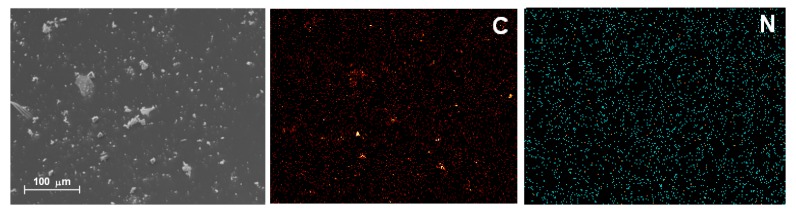
SEM image and carbon (**C**) and nitrogen (**N**) EDS mapping of the xerogel with the H_2_T(*p-*COOH)PP species bonded using NAEPTES as a bridges and inside pores of silica modified with dodecyl groups.

For simplicity, Scanning Electron Microscopy (SEM) was employed to visualize the aspect of the samples that display the best absorption and fluorescence spectra. These samples were those in which the H_2_T(*p-*COOH)PP species was covalently bonded by means of APTES (H_2_P-APTES) to a SiO_2_ network modified with phenyl groups or by using NAEPTES ((H_2_P-NAEPTES) and bonded to a silica matrix modified with docedyl groups. For brevity, we show here images of this last sample (Figure 6), but in both cases, the SEM images reveal soft surfaces, without evident textural details. Besides, in the same samples there exist an homogeneous carbon and nitrogen atom distribution which can be associated with the presence of organic groups attached to the silica network and with the H_2_T(p-COOH)PP macrocycle trapped in the network.

### 2.5. Textural Characterization

N_2 _sorption-desorption experiments were carried out at 76 K in order to determine the textural characteristics of the organo-modified silica networks; additionally, these parameters can help to elucidate the hypothetical situation of the H_2_T(*p-*COOH)PP species when bonded to the pore network walls through APTES or NAEPTES. The ensuing N_2_ sorption-desorption isotherms of both sets of samples (see Supplementary figures) were all IUPAC Type I isotherms [45,46] with H3 hysteresis cycles [47]. The narrow hysteresis cycle observed for most of the cases, suggest that both condensation and evaporation of N_2_ molecules occur easily without the intervention of cooperative phenomena or pore blocking effects.

However, in the case of samples synthesized with APTES and with DoTEOS, AmyTEOS or without the involvement of an OSA compound (blank-b), the hysteresis cycle resulted wider. A similar situation was observed in the case of samples synthesized with NAEPTES, AmyTEOS, and EtTEOS as well as for blank-a structure. This situation could be attributed to a more complicated (not straightforward) desorption process because of the existence of narrow interconnecting necks between pore cavities in the network. All this means that a pore blocking effect starts arising in the above- mentioned specimens.

Additionally, in both sets of samples the total sorption capacity differed from that observed for the pristine silica network (blank-a), blank-b, or blank-c. The total pore volume resulted higher or lower in the different samples; these differences suggest that the existence and identity of alkyl or aryl groups that are attached to the pore network modify the total sorption capacity (*i.e.*, calculated from the upper plateau of the sorption isotherm) of the xerogels. This effect could be credited to repulsive or attractive interactions between the porphyrin bonded to the pore network and the alkyl or aryl groups existing at the pore walls, effects which induce the expansion or contraction of the pore cavity that is created around the macrocycle during the sol-gel process.

**Figure 7:**
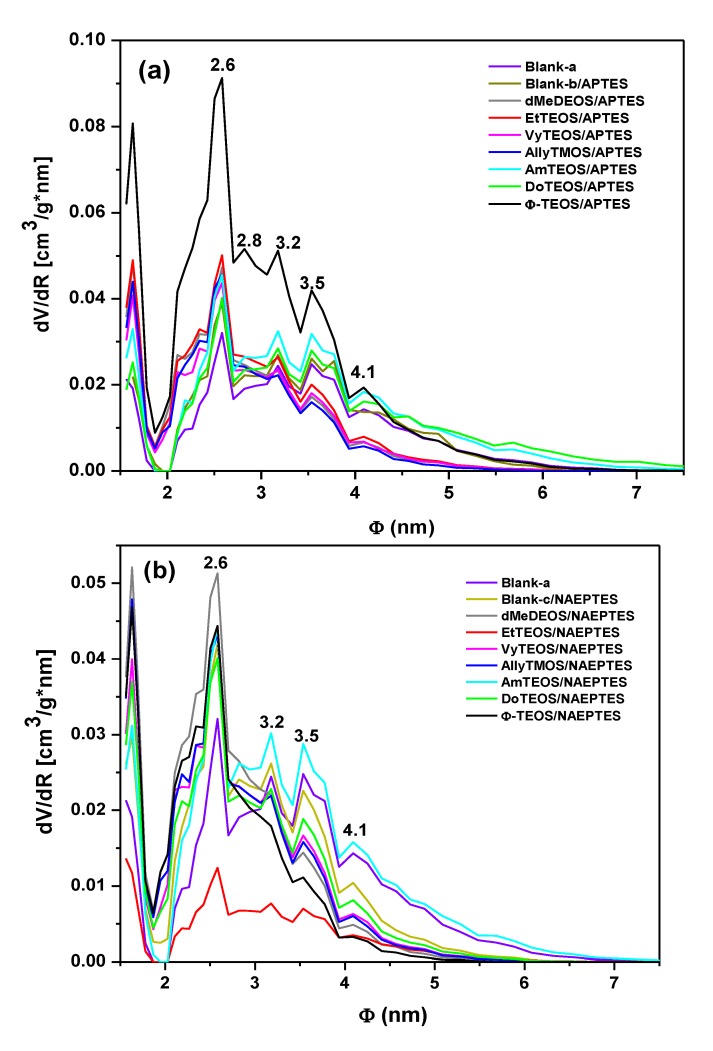
Pore-size distribution (PSD) calculated by assuming spherical pore cavities, from the N_2_ adsorption isotherms of organo-modified silica xerogel networks with H_2_T(*p*-COOH)PP species covalently bonded to the pore walls through the linking action of (**a**) APTES or (**b**) NAEPTES.

From the boundary adsorption curve while assuming spherical pore cavities, the pore-size distribution (PSD) of all the samples were calculated (Figure 7). Interesting observations can be made about these PSD results, starting from the fact that the shapes of all these curves resulted very similar to each other. A most intense peak corresponding to 2.6 nm of width can be seen for all specimens. One can imagine that the inserted macroycle molecules are wrapped inside a xerogel matrix made of pore cavities of different sizes that extends from that value to 7.0 nm, approximately. The larger pore widths were determined for the blank-b and samples containing phenyl (Φ-TEOS) or ethyl (EtTEOS) groups for the set of specimens synthesized with APTES. In the case of the samples obtained using NAEPTES, the higher pore widths were observed for dMeDEOS, AllyTMOS, and blank-c xerogels. Interestingly, the most intense fluorescence was observed in systems in which the porphyrin was bonded through APTES to a pore network modified with phenyl groups. In the case where NAEPTES was chosen, better results were obtained when the silica network was modified via dodecyl or allyl groups. Again, since all the samples were synthesized by means of the same molar mixture, the above textural differences could only be associated to the alkyl or aryl group that is attached to the network and which arise from the OSA compound involved in the preparation.

The average pore diameter (Φ) and the specific surface area of each substrate were calculated from the corresponding treatment of the N_2_ isotherms (Table 1). In both sets of samples, the presence of the trapped porphyrin and of alkyl or aryl groups induces the creation of larger specific surface areas than those determined in the pristine silica network (blank-a). The average pore diameters (Φ) determined for the blank-a, and blank–b substrates, were similar to each other. The diameters of the SiO_2_ cavities formed around the H_2_T(*p*-COOH)PP species ranged from 2.33 nm to 3.73 nm. In the particular case of the samples synthesized using APTES, the existence of dodecyl or amyl groups (3.73 and 3.47 nm) attached to the pore walls induces the creation of larger pore sizes, if compared to those obtained in the blank-a or blank-b systems. This event could be attributed to repulsive interactions between these long organic chains and the bonded porphyrin. Smaller pore diameters were determined for samples in which short organic chains such as methyl or ethyl groups, or chains including double bonds such as vinyl, allyl or phenyl groups, were attached to the silica matrix. This last event could be attributed to the closest approximation of these groups to the porphyrin ring and to the attractive interaction between double bonds. Contradictorily to the presupposed result, smaller width sizes were obtained when NAEPTES was used to fix the porphyrin to the network, in the presence or absence of other organic groups attached to the pore surfaces. In the latter set of samples, the use of NAEPTES restricts the pore sizes (to around 3.00 nm) thus causing the organic groups attached to the network to remain closer to the porphyrin macrocycle while staying situated at the proximity of the macrocycle center and, at both sides of the molecular plane (Scheme 1a,b). That is, the establishment of longer unions is not a sufficient guarantee for the formation of large cavities around the porphyrin molecule. Additionally, the establish of those longer bonds render no better fluorescent intensity, this is possibly because these unions localized in the same molecular plane, something which facilitates the approximation of Si-OH or organic groups to the center of the macrocycle, then inhibiting the occurrence of the emission process. By using NAEPTES as bridging species, a better fluorescence emission was observed when dodecyl or allyl groups were attached to the silica network while the average pore width determined for these samples were of 2.89 and 2.75 nm, respectively. However, the large size of the precursory species (Scheme 1b) and the presence of long chains, such as dodecyl groups suggest a very compact array of molecular species inside the pores.

**Table 1:** Specific Surface areas and average pore widths (Φ) of organo-modified silica xerogels in which H_2_T(*p*-COOH)PP species are covalently bonded through the use of APTES or NAEPTES.

	APTES			NAEPTES		
sample	Specific Surface Area (m^2^/g)	Pore Volume (cm^3^/g)	Average pore diameter, Φ (nm)	Specific Surface Area (m^2^/g)	Pore Volume (cm^3^/g)	Average pore diameter, Φ (nm)
Blank-a *^a^*	544.0	0.331	3.43 ± 1.01			
Blank-b*^a^*	686.3	0.430	3.34 ± 0.81			
Blank-c*^a^*				643.9	0.37	3.02 ± 0.70
dMeDEOS	700.4	0.380	2.77 ± 0.65	703.3	0.37	2.68 ± 0.52
EtTEOS	775.1	0.425	2.81 ± 0.64	763.6	0.44	3.43 ± 1.01
VyTEOS	623.9	0.353	2.33 ± 0.88	634.4	0.34	2.80 ± 0.60
AllyTMOS	668.0	0.360	2.75 ± 0.56	662.7	0.36	2.75 ± 0.60
AmTEOS	804.3	0.539	3.47 ± 1.28	751.8	0.48	3.35 ± 1.12
DoTEOS	745.7	0.530	3.73 ± 1.74	619.5	0.34	2.89 ± 0.69
Φ-TEOS	675.7	0.400	2.96 ± 0.81	657.4	0.33	2.61 ± 0.45

*^a^* Blank-a included neither porphyrin nor OSA compounds. Blank-b and blank-c samples were synthesized without OSA compound and bonding the porphyrin by using APTES or NAEPTES, respectively. ± define the standard deviation determined from the pore widths distributions.

### 2.6. Situation of the H_2_T(p-COOH)PP Species inside the Pores

**Figure 8:**
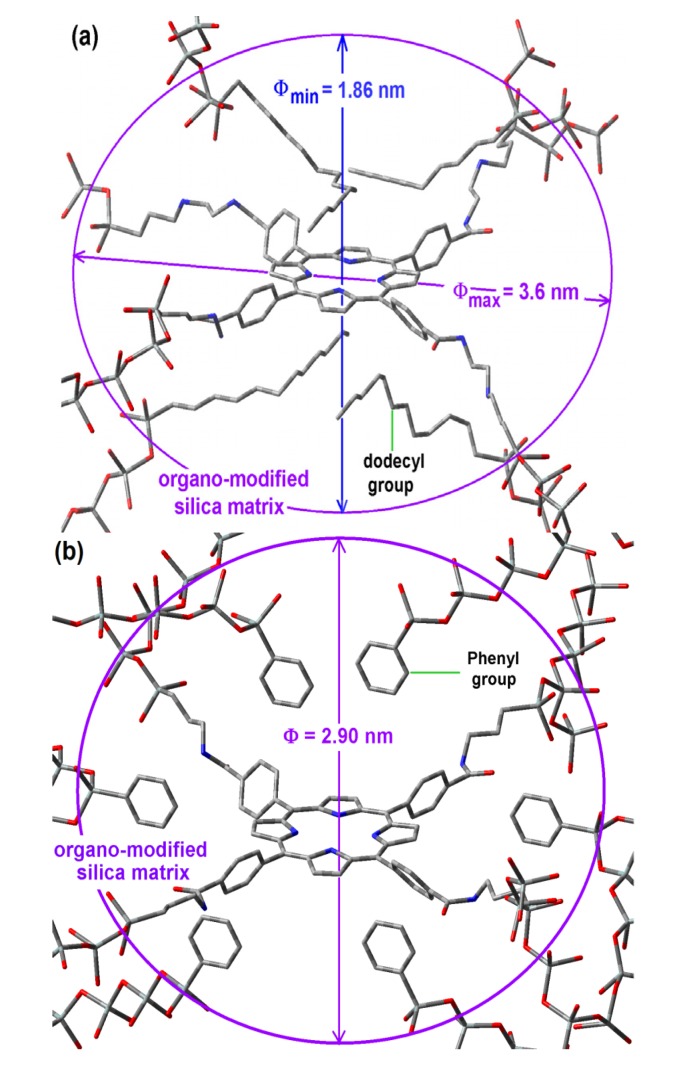
Hypothetical structure of a pore cavity containing the H_2_T(*p-*COOH)PP species covalently bonded, through the use of NAEPTES to the pore walls of silica modified with (**a**) *dodecyl* groups (Do) or (**b**) through the use of APTES and modified with *phenyl* groups (Φ).

Considering that the formation of the cavities around the precursory trapped species (*i.e.*, the macrocycle with its bridging chains) is determined by its size and its interactions with those groups able to remain closer and oriented toward it during the xerogel network creation, the shape of the resultant cavity is determined by both events. If this is true, the pore widths determined from a spherical model and associated to the sorption-desorption of N_2_, could be imputed to a different pore shape: ellipsoids, rather than spheroidal cavities. In the case of samples synthesized with NAEPTES, the pore volume calculated to a given diameter (Φ) can be instead associated to an ellipsoidal cavity, in which the larger semiaxis corresponds to the length of the precursory species (Scheme 1b), while its shorter semiaxis is being determined by the attractive or repulsive interactions between the porphyrin and the groups localized over and below of the molecular plane. In the particular case of the sample with dodecyl groups attached to the pore network, the hypothetical longer axis can correspond to the length existing between two opposite silicon atoms in the respective precursors (Φ_max_ = 3.60 nm) while the shorter width (Φ_min_), could be calculated from the volume associated to a sphere of diameter 2.89 nm (Table 1), *i.e.*, 1.86 nm (Figure 8a). Inside these cavities, dodecyl groups can be localized at both sides of the molecular plane.

For the case of samples in which the H_2_T(*p-*COOH)PP species was bonded to the pore walls via APTES, the above consideration induces us to assume that pores are more likely to be considered as spherical cavities (Figure 8b). Thus, the optimization of fluorescence may be result of the establishment of unions between the porphyrin and the pore network together with the existence of adequate organic chains around the trapped molecule.

The above results indicate that, in a first instance, the bridging species used to bond the porphyrin to the pore network determine the size of the pore formed around it, however, the repulsive and attractive interactions of the macrocycle with the organic groups attached to the pore walls modulate the final size and induce a lowly polar physicochemical environment inside the formed cavities. Some groups such as phenyl, dodecyl, and allyl groups induce a more convenient polarity inside the pores in order to make easier the occurrence of electronic transitions of the macrocycle molecule, which happen inside a silica network in similar way as those that occur in a solution of the free molecule.

### 2.7. Application of the Methodology Developed from Encapsulation of the H_2_T(o-NH_2_)PP Species

**Figure 9:**
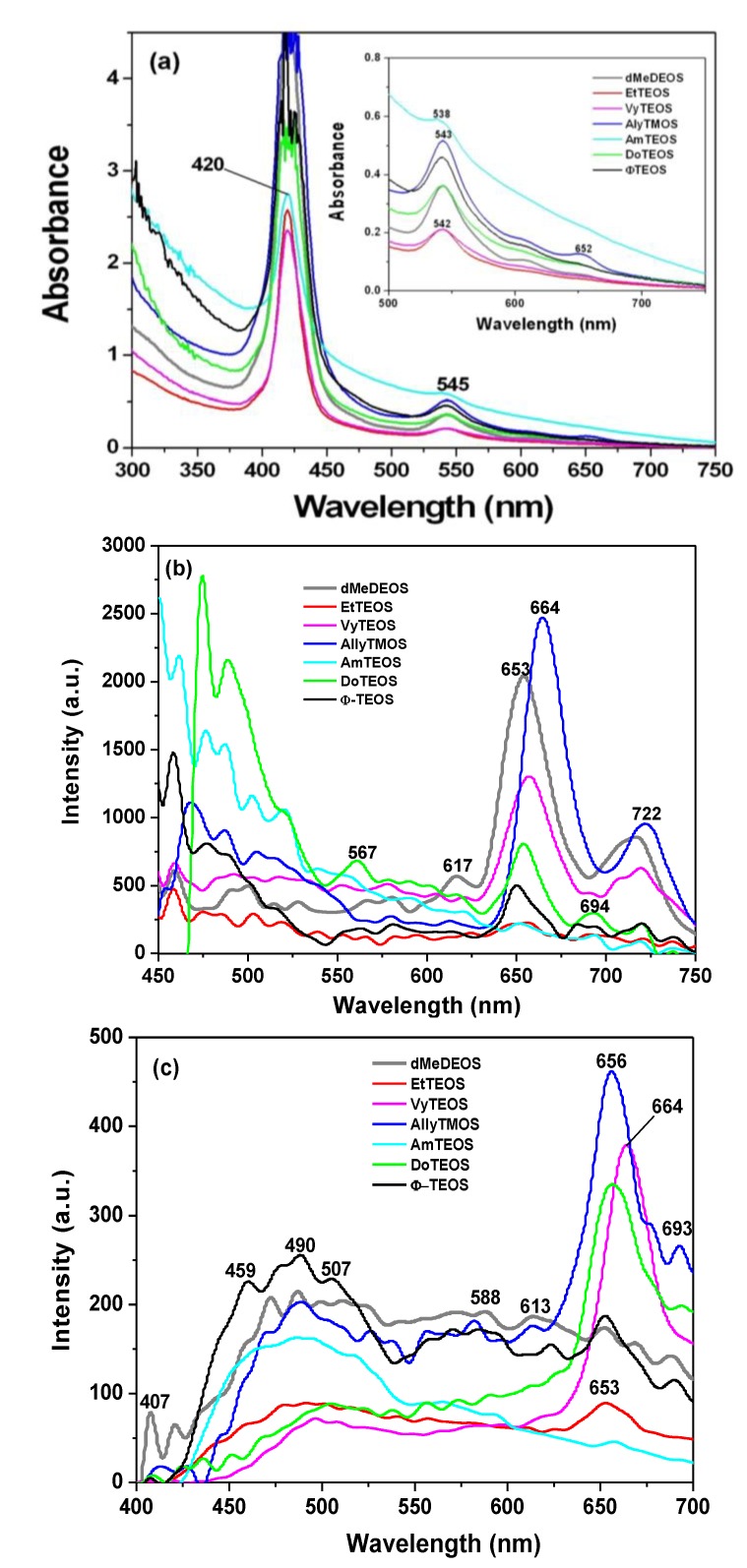
(**a**) Absorption spectra of H_2_T(*o-*NH_2_)PP species covalently bonded to the pore walls of silica modified with organic groups and the respective fluorescence spectra resulting from using excitation light of (**b**) 420 nm and (**c**) 370 nm.

In order to apply the developed encapsulation methodology for preserving and optimizing the fluorescence of a porphyrin containing substituents located outside the molecular plane, the H_2_T(*o-*NH_2_)PP species was bonded to the pores of organomodified silica. The samples were synthesized by using the same molar mixtures, as in the case of the H_2_T(*p-COOH*)PP species, to fix these macrocyclic species inside the pores of organomodified silica, but employing IPTES as chemical bridges (Scheme 1c). For simplicity, the only analyses made for this set of samples correspond to absorption and fluorescence experiments. In the respective absorption spectra of these xerogels, the high intensity Soret bands were observed at around 420 nm (Figure 9a).

In the same spectra, the Q_IV_ signal was not observed, while the Q_III_, Q_II_, and Q_I_ bands were localized at around 543, 610 and 652 nm, respectively (Figure 9a insert). This change in the Q bands pathway in the absorption spectrum of the H_2_T(*o-*NH_2_)PP solution could be attributed to the interactions of the macrocycle with the silica network, something that inhibits the vibrational coupling that causes the surge of the Q_IV_ band or also as a consequence of the change in polarity around the trapped macrocycle.

In all cases, the intensity of the fluorescent signals apparently depends on the identity of the organic group attached to the network; the emission decreasing in the following order: allyl > methyl > phenyl > docecyl > amyl > ethyl > vinyl (Figure 9a). These results suggest that allyl, methyl, and docecyl chains induce a more convenient physicochemical environment around the bonded porphyrin, probably because of their very close approximation to it.

By using an excitation wavelength (λ_exc_) of 420 nm (Figure 9b), the bands pathway in the range from 600 to 750 nm, is more similar to that observed for H_2_T(*o-*NH_2_)PP species in solution than if using light of 370 nm (Figure 9c). In the case of these latter spectra, the most intense bands were localized at around 653 to 664 nm, while a shallow band appears at around 700 nm. The most intense bands were observed in the case of the sample modified with allyl, vinyl, and dodecyl groups (Figure 9c). However, some other bands of a lower intensity were observed from 400 to 600 nm, attributed to the silica matrix and/or to the interactions of the macrocycle with the surface groups attached to the organo-modified silica network, such as alkyl, aryl and remnant Si-OH groups. The shape, number and localization of bands observed in this region for the set of samples in which H_2_T(*o-NH_2_*)PP species was bonded to the pore network differ from those when H_2_T(*p*-COOH)PP species were trapped. This observation suggests that interactions between porphyrin and organomodified silica matrices results more intense with H_2_T(*o-*NH_2_)PP species, certainly because of its own structure.

Furthermore, the intensity of the signals appearing from 450 to 600 nm depended on the identity of the OSA compound used; this situation is more evident when fluorescence spectra were obtained by employing an excitation light of 420 nm (Figure 9b). However, a sharper set of bands, attributed to the trapped macrocycle, was observed at around 651 to 664 nm and 706 to 722 nm. The best results were obtained when allyl, methyl, vinyl, dodecyl and phenyl groups were attached to the walls of the silica network.

All the above fluorescence results complement the absorption spectroscopy analysis and confirm the hypothesis that the presence and proximity of groups such as allyl, methyl, dodecyl, and phenyl induce a convenient physicochemical environment around the H_2_T(*o-*NH_2_)PP species. Additionally, these results could mean that some groups can remain closer of the macrocycle without interfering with its luminescence properties. Furthermore, is possible that some of the used alkyl or aryl groups can act as a shield against the deleterious (*i.e.*, aggregation) effect caused by the interactions of the macrocycle with the surface Si-OH groups.

**Figure 10:**
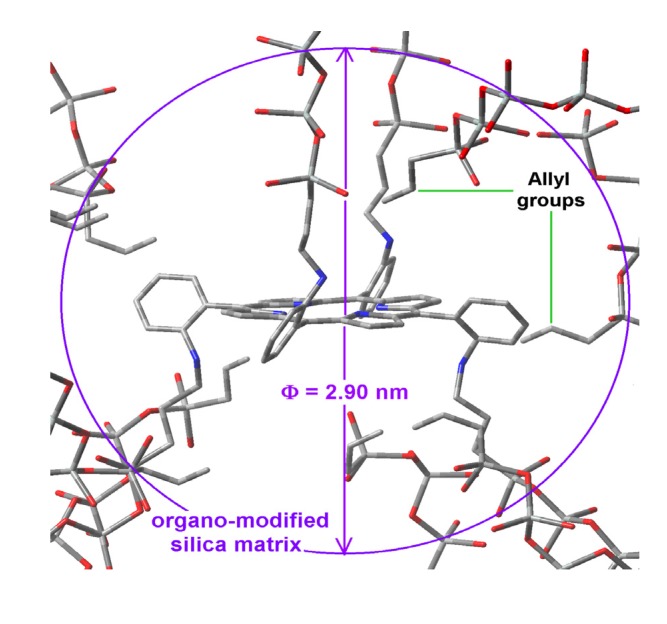
Hypothetical structure of a pore containing the H_2_T(*o-*NH_2_)PP species covalently bonded through the use of IPTES to the pore walls of silica modified with allyl groups.

Surely, the amide or urethane groups that are connecting the silica network to the H_2_T(*p-*COOH)PP or H_2_T(*o-*NH_2_)PP species, respectively (Scheme 1), interact in quite different ways with the surface groups of the organomodified silica network. In all cases, the last fluorescence results, especially those obtained using 370 nm wavelength demonstrate that the preservation of fluorescence emission results are more complicated for the H_2_T(*o-*NH_2_)PP species than for the H_2_T(*p-*COOH)PP compound. This difference can be due to the stronger interactions of the first species with organomodified silica networks, definitely because of the different localization of the peripheral substituents in each compound (*i.e.*, the positions of the –NH_2_ or the COOH groups, respectively) from which the bridges for fix these species to the pore network were formed. These unions are localized on the same molecular plane as in the case of the H_2_T(*p-*COOH)PP (Scheme 1a,b and Figure 8) and at opposite sides of the molecular plane in the case of the H_2_T(*o-*NH_2_)PP species (Figure 10). This last situation caused a more difficult approximation (over or below) of alkyl or aryl groups to the central regions of the macrocycle. Then, these organic groups are likely to be localized at the periphery far from the center of the macrocycle. Considering the results obtained with the H_2_T(p-COOH)PP species, it is possible to suppose that, in a similar way the sizes of the cavities formed around the H_2_T(*o-*NH_2_)PP species were determined by the length of its unions existing above and below of the molecular plane and also by its interactions with groups that remain during the duration of the sol-gel process (Figure 10).

All the above mentioned results evidence that, the establishment of longer molecular unions provides no guarantee for attenuating the interactions of surface groups with the trapped macrocycle, something which interferes with the display of its electronic transitions. As shown, the establishment of longer covalent unions arising from the use of NAEPTES as bridging species produces smaller pore sizes than those obtained with APTES. This comparison suggests that, the occurrence of attractive interactions between the organic groups (attached to the silica network) and the central regions of the porphyrin induces the formation of ellipsoidal cavities with the consequent interference effect over the electronic transitions of the trapped macrocycle. On the other hand, when the interaction with the center of the porphyrins is inhibited by the establishment of covalent unions created on opposite sides of the molecular plane, the approximation of the attached organic chains could more probably happen at the periphery and in the proximity of the macrocycle plane. That is, the spatial localization of the substituents of the free bases of the porphyrin plays an important role in the modulation of the interactions that inhibit the efficient display of its physicochemical properties. Furthermore, the adequate selection of these substituents and its spatial localization around the macrocyclic compounds can determine the formation of cavities of different shapes and sizes. In this sense, the optimization of fluorescence involves an authentic design of cavities as well as an adequate disposition of the different chemical species around the trapped species.

We consider that the results shown herein demonstrate the possibility of optimizing the displaying of the optical properties that tetrapyrrole macrocycles show in solution but now incorporated in solid hybrid systems. This efficient displaying can be associated to the stability and unaltered chemical situation of the porphyrin inside the silica pores. Consequently, the above mentioned methodology can be employed to trap, inside hybrid solid systems, diverse tetrapyrrole macrocyclic species, as well as other kind of active molecules in order to optimize the properties that display in solution, such as the catalytic, coordination, sensoring or some other properties. Furthermore, the possibility of synthesizing similar systems from TiO_2_, ZrO_2_ or Al_2_O_3_ networks is now being explored by our research group. Eventually, these hybrid systems should assure very interesting and useful technological applications. 

## 3. Experimental

### 3.1. General Information

All metal alkoxides and solvents employed in this work were acquired from Aldrich and Fluka (Sigma-Aldrich Química, S.A. de C.V., Toluca, México).

### 3.2. Chemistry

*ortho-* and *para-*Substituted tetraphenylporphyrins, H_2_T*(o-S or p-S*)PP (Scheme 1), were synthesized from pyrrole and the respective substituted benzaldehyde by the Rothemund reaction [48] and the Adler method [49,50]. The as-prepared solid was washed sequentially, with chloroform, acetone, and ethanol, and further purified through a chromatographic silica column employing chloroform and ethanol as eluents. The final solid was characterized by absorbance and fluorescence spectroscopies in the UV-VIS-NIR range, as well as by elemental qualitative analysis.

To obtain translucent monolithic organosubstituted silica xerogels, including H_2_T(*o-*NH_2_ or *p-*COOH)PP species bonded to the pore network, a two step route was followed. In the first step, the respective precursors, H_2_P-FA (Scheme 1), were synthesized from a 1:4 molar mixture of porphyrin and the convenient functionalized alkoxide (FA); this mixture was taken 6 h to reflux in ethanol under a N_2_ atmosphere. In all cases, the reaction was followed by FTIR spectroscopy. Due to the presence of carboxyl groups in H_2_T(*p-*COOH)PP species, functionalized alkoxides*, i.e.*, either 3-aminopropyl-triethoxysilane (APTES) or 3-*N*-(2-aminoethylamino)propyltrimethoxysilane (NAEPTES) were used for establishing amide unions arising from amine groups of the alkoxide and carboxyl groups in the porphyrin (Scheme 1a,b). 3-Isocyanopropyltriethoxysilane (IPTES) was chosen for reacting with the amine groups at the *ortho* positions of H_2_T(*o-*NH_2_)PP species (Scheme 1c).

In the second step, the respective precursor (H_2_P-APTES, H_2_P-NAEPTES or H_2_P'-IPTES) was reacted with mixtures of triethoxysilane (TEOS) and diverse organosubstituted alkoxides (OSA), such as, dimethyldimethoxysilane (dMeDEOS), vinyltriethoxysilane (VyTEOS), ethyltriethoxysilane (EtTEOS), allyltrimethoxysilane (AllyTMOS), amyltriethoxysilane (AmyTEOS), dodecyl-tetiethoxysilane (DoTEOS), and phenyltriethoxysilane (Φ-TEOS) (Scheme 2). The precursor molecule was included together with a H_2_O:TEOS + OSA:HCl:H_2_-P-FA molar ratio mixture equivalent to 19.6:1:10^−3^:10^−3^–10^−6^, which rendered a final V_f_ total volume [24,25,26,27,28]. In all these mixtures, only 1% v/V_f_ of OSA compound was employed in order to render translucent SiO_2_ xerogels**. **Additionally, three samples were synthesized in the absence of OSA compounds for being employed as references: (i) a pristine silica network (blank-a) was prepared from the same molar ratio as mentioned above; (ii) blank-b and blank-c were synthesized by bonding the porphyrin molecule to the silica pore walls through APTES or NAEPTES, respectively.

The above mixtures were poured inside closed plastic cells, and the gelling process was monitored through absorption spectroscopy. When the gel contraction step ended, samples were dried for three weeks at room temperature; afterward for three days at 70 °C, and finally for one day at 125 °C. The samples were characterized by FTIR (Perkin-Elmer GX instrument, Perkin-Elmer Inc., Waltham, MA, USA), absorption (Cary-Varian 500E spectrophotometer, Varian Optical Spectroscopy Instruments, Mulgrave, Victoria, Australia), ^29^Si-NMR (JEOL ECLIPSE + 300 spectrometer at 300 MHz, JEOL USA, Peabody, MA, USA), fluorescence spectroscopy (Perkin-Elmer LS5 spectrofluorometer, Perkin-Elmer Inc., Waltham, MA, USA for solid samples and ISS PC1 spectrofluorometer, Champaign, IL, USA, for samples in solution) as well as by SEM, powder X-ray diffraction (Instruments D-500, Siemens Analytical X-Ray Instruments Inc. Karlsruhe, Germany), and N_2_ sorption at 76 K (automatic volumetric Quantachrome autopore 1L-C instrument, Quantachrome Instruments, Boynton Beach, FL, USA).

The pore widths of the silica matrix containing macrocyclic species bonded to the xerogel network were calculated by the NLDFT approach applied to N_2_ adsorption branch of the isotherms [45,46] while assuming spherical pore cavities.

## 4. Conclusions

All the above results and analyses prove that the postulated synthetic methodologies make possible the covalent bonding of free bases of porphyrins, in stable and monomeric form inside metal oxide pore networks.

Combined with the chemical fixing of the porphyrins, the attachment of alkyl or aryl groups on the pore walls of silica networks induces a lower polar environment inside the void cavities that are created around the trapped macrocycle. This modified environment, combined with the repulsive or attractive interactions, between the porphyrin and the organic chains attached to the solid surface causes that the absorbance and emission of light by the macrocycle can take place in a similar fashion as that occurring in a solution of the free macrocyclic molecule.

As it has been proved herein, the establishment of long chemical unions is not sufficient to inhibit the interactions of the macrocycle with the surface groups anchored at the pore walls. In all cases, the occurrence of attractive interactions between these groups and the center of the macrocycle can possibly cause the formation of ellipsoidal, rather than spherical cavities, around the tetrapyrrolic molecule with the consequent interfering effect over the electronic transitions of the trapped macrocycle (*i.e.*, luminescence properties). Likewise, the spatial distribution of peripheral substituents around the macrocyclic molecule indeed affects indeed the way by which the tetrapyrrolic species interacts with the surface groups attached to the pore walls, and most than all also affects the efficient displaying of the porphyrin properties.

Further work could consider to assume the pore cavities created around trapped tetrapyrrole compounds as being of an elliptical instead of spherical shape in order to calculate in a more precise (although more complicated) way the incumbent pore-size distributions. In all cases, the covalent bonding of macrocycle species and its immersion inside a less polar environment convey into a satisfactory strategy to preserve, in a solid system, the absorbance, emission, and other interesting physicochemical properties that free bases of porhyrins display in solution. The optimizations of these properties constitute an authentic design of cavities and, an assurance for creating useful devices for *avant garde* applications in optical, catalytic, sensoring, and medical devices.

## Figures and Tables

**Figure A1:**
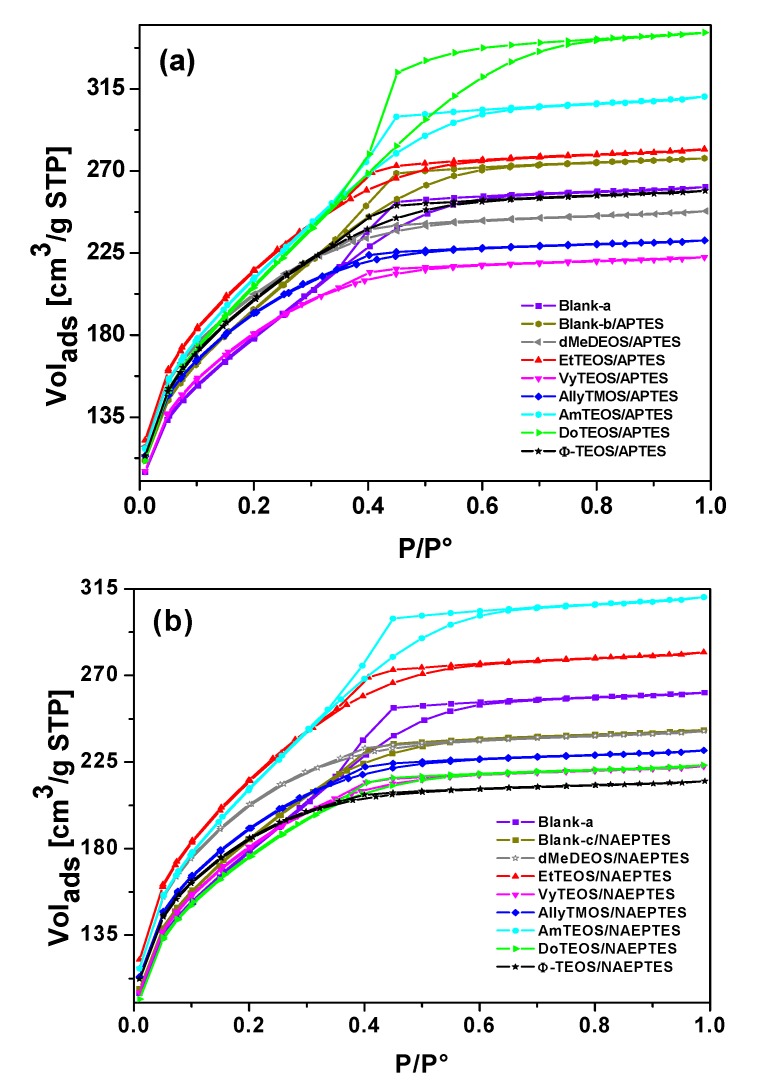
N_2_ sorption-desorption isotherms at 76K of organo-modified silica pore networks including H_2_T(*p-*COOH)PP species bonded to the walls through the use of APTES (**a**) or NAEPTES (**b**).
